# Overexpression of *PdC3H17* Confers Tolerance to Drought Stress Depending on Its CCCH Domain in *Populus*

**DOI:** 10.3389/fpls.2019.01748

**Published:** 2020-01-24

**Authors:** Yamei Zhuang, Congpeng Wang, Yang Zhang, Sihui Chen, Dian Wang, Qing Liu, Gongke Zhou, Guohua Chai

**Affiliations:** ^1^University of Chinese Academy of Sciences, Beijing, China; ^2^Key Laboratory of Biofuels, Chinese Academy of Sciences, Shandong Provincial Key Laboratory of Energy Genetics, Qingdao Institute of Bioenergy and Bioprocess Technology, Chinese Academy of Sciences, Qingdao, China; ^3^College of Resources and Environment, Qingdao Agricultural University, Qingdao, China

**Keywords:** the CCCH domain, *Pd*C3H17, drought tolerance, ROS scavenging activity, xylem vessel cell number, *Populus*

## Abstract

Plant CCCH zinc finger proteins control growth, development, and stress responses mainly at the post-transcriptional level. Currently, limited reports are available about the roles of plant CCCH proteins in drought tolerance. In this study, we provided evidence showing that *Pd*C3H17 from *Populus deltoides × P. euramericana* involves drought tolerance and response. Overexpression of *PdC3H17* in poplar caused dwarf, resulted in higher stem water potential, and showed increased photosynthetic and ROS-scavenging abilities, thereby enhancing tolerance to drought stress, compared to controls. Accordingly, after drought treatment the stem elongation and thickening rates of these overexpression lines were higher than those of the controls. However, overexpression of the coding region excluding the CCCH domain of *Pd*C3H17 roughly exhibited WT-like physiological and drought-resistant phenotypes, indicating the requirement of the CCCH domain for *Pd*C3H17 controlling these processes. In addition, N-terminal sequence of *Pd*C3H17 was found to possess transcriptional activity ability in yeast cells. Together, our results suggest that *Pd*C3H17 may depend on its CCCH domain to control drought tolerance in *Populus*.

## Introduction

Poplar (*Populus* spp.), a fast-growing tree species, is widely used for timber, pulp, and paper, and has potential as a source of bioenergy (Du and Groover, 2010). Poplars are drought-sensitive woody species and have evolved versatile mechanisms to mitigate drought stress, including reducing transpiration, scavenging reactive oxygen species (ROS), generating abscisic acid (ABA), and altering plant morphology (Harfouche et al., 2014). Accumulating evidence in *Populus* shows that a large amount of genes participate in controlling these processes under drought conditions. For example, *Pd*EPF1, a member of the epidermal patterning factor (EPF) family in *Populus nigra* × (*Populus deltoides* × *Populus nigra*), regulates water use efficiency and drought tolerance by modulating stomatal density (Wang et al., 2016). *Pe*CHYR1, an ubiquitin E3 ligase in *Populus euphratica*, enhances drought tolerance *via* ABA-induced stomatal closure by ROS production (He et al., 2018). The AREB1 transcription factor influences histone acetylation of *Ptr*NAC006, *Ptr*NAC007, and *Ptr*NAC120, resulting in key physiological alterations conducive to drought tolerance and resilience and thereby changing drought responses in *Populus trichocarpa* (Li et al., 2019). Currently, it remains unclear that the regulatory mechanisms underlying drought response and tolerance in tree species.

The CCCH zinc finger family contains a typical C3H-type motif and members of this family had already been identified in organisms from yeast to human (Chai et al., 2015). Plant CCCH proteins play vital roles in a wide variety of growth, development, and stress responses, and may perform both transcriptional and posttranscriptional regulation (Bogamuwa and Jang, 2014). In *Arabidopsis*, the subfamily IX members of CCCH proteins are shown to mediate stress signaling based on qRT-PCR analysis (Wang et al., 2008). This is validated by subsequent functional analyses. AtTZF1/AtC3H23 functions as a regulator of ABA- and GA-mediated growth and drought response (Lin et al., 2011). Overexpression of AtTZF2/AtC3H20 or AtTZF3/AtC3H49, the homologs of AtTZF1, improves ABA hypersensitivity, reduces transpiration, and enhances drought tolerance (Lee et al., 2012). OsTZF1 is an ortholog of AtTZF1 in rice and confers tolerance to drought and high-salt stresses by regulating stress-related genes (Jan et al., 2013). OsC3H47 decreases ABA sensitivity and promotes drought and salt tolerance in rice seedlings (Wang et al., 2015). GhTZF1, an ortholog of AtTZF1 in cotton, regulates drought stress responses and delays leaf senescence by inhibiting ROS accumulation in transgenic *Arabidopsis* (Zhou et al., 2014). Recently, a non-tandem CCCH protein, IbC3H18, is shown to act as a nuclear transcriptional activator and enhances salt, drought, and oxidation stress tolerance in sweet potato (Zhang et al., 2019), suggesting that in addition to subfamily IX, the members of other CCCH subfamilies in *Arabidopsis* may participate in controlling drought tolerance.

Our previous study demonstrated that *Populus* contains 91 *CCCH* gene family members, 90% of which are physically distributed on the duplicated blocks (Chai et al., 2012). Thirty-four paralogous pairs are identified in these CCCHs, of which 22 pairs (65%) may be created by the whole genome segment duplication. Of them, *Pd*C3H17 and *Pd*C3H18, a pair of paralogs, are direct targets of *Pd*MYB3 and *Pd*MYB21, which are second-level master switches of the transcription network for wood formation (Ye and Zhong, 2015), and function as positive regulators of secondary xylem development in both *Arabidopsis* and poplar (Chai et al., 2014). *Pd*C3H17 has two orthologs (AtC3H14 and AtC3H15, belonging to subfamily II in CCCHs) in *Arabidopsis* (Wang et al., 2008; Chai et al., 2014). AtC3H14 and AtC3H15 redundantly control stem elongation and secondary cell wall thickening as well as anther development (Kim et al., 2014; Chai et al., 2015). However, the roles of the CCCH proteins in response to environmental stresses in trees have not yet been elucidated.

In this study, we provided evidence showing that *Pd*C3H17 controls drought response depending on its CCCH domain in a hybrid poplar. Overexpression of *PdC3H17* in poplar resulted in stronger drought-tolerance phenotype, correlating with a significant increase in both leaf ROS-scavenging abilities and stem xylem vessel cell numbers, compared to control plants. However, transgenic poplars overexpressing the coding region excluding the CCCH domain of *Pd*C3H17 roughly exhibited wild type-like phenotypes. Combined with other biochemical evidence, we suggest that *Pd*C3H17 may be a novel regulator of drought tolerance in poplar.

## Materials and Methods

### Transcriptional Activation Assay in Yeast

**Figure 1:**
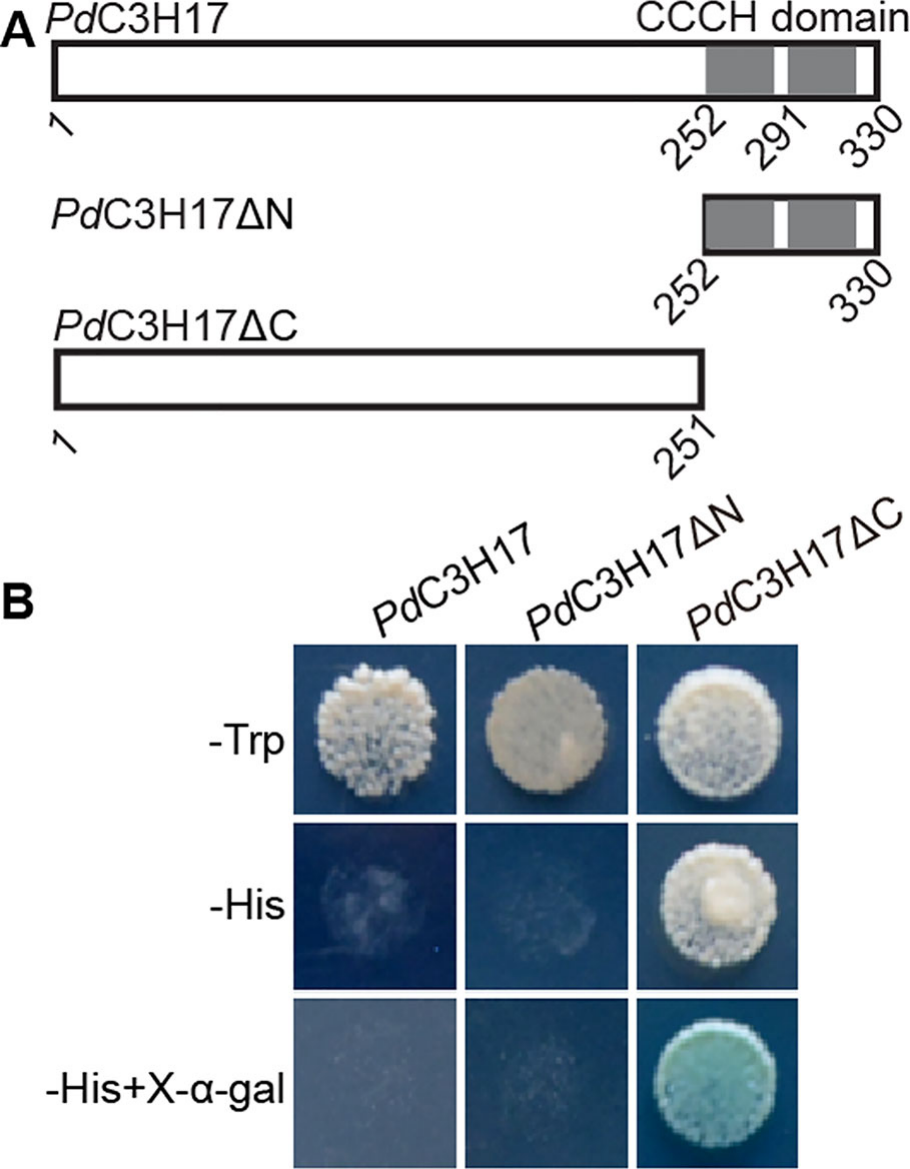
Transactivation analysis of different regions of *Pd*C3H17 fused with the GAL4 DNA-binding domain in yeast. **(A)** Diagram of *Pd*C3H17, *Pd*C3H17ΔN, and *Pd*C3H17ΔC proteins. **(B)** Only *Pd*C3H17ΔC is able to activate the expression of the *HIS3* reporter gene.

The full-length coding region (*Pd*C3H17) and the fragments including (*Pd*C3H17ΔN) or excluding (*Pd*C3H17ΔC) the CCCH domain of *PdC3H17* (**Figure 1A**) were separately fused in frame with the GAL4 DNA-binding domain in pGBKT7 (Clontech) using the appropriate primers containing the *EcoR*I site. *Pd*C3H17: (Forward, 5′-ATGGAGGCCGAATTCATGGAGAAAACAGAATCACCA-3′; Reverse, 5′-GATCCCCGGGAATTCTCAACGAGGACCCAGCAGTAACC-3′). *Pd*C3H17ΔC: (Forward, 5′-ATGGAGGCCGAATTCATGGAGAAAACAGAATCACCA-3′; Reverse, 5′-GATCCCCGGGAATTCCGTGAAGGTTTTGGCGGGAA-3′). *Pd*C3H17ΔN: (Forward, 5′-ATGGAGGCCGAATTCATGCAAGGGATGTGGAAGACAG-3′; Reverse, 5′-GATCCCCGGGAATTCGTCAGTGAGGGAGTGGCGAAA-3′). The recombinant vectors and the pGBKT7 empty vector were transformed into yeast strain AH109. The yeast liquid cultures were dropped on the synthetic dropout (SD)/Trp‐ and SD/Trp‐/His‐/Ade (adenine)‐agar media. The transcriptional activation activity of each protein was evaluated according to their growth status and the activity of α-galactosidase.

### Vector Construction and Plant Transformation

Full-length *Pd*C3H17 and *Pd*C3H17ΔC were separately ligated downstream of the 35S promoter in pCAMBIA1300-GFP vector to generate the overexpression constructs. Primers for *PdC3H17* were (Forward, 5′-CGGGGTACCATGGAGAAAACAGAATCACCA-3′, the *Kpn*I site marked with underline) and (Reverse, 5′-CGGGATCCTCAACGAGGACCCAGCAGTAACC-3′, the *BamH*I site). Primers for *PdC3H17ΔC* were (Forward, 5′-CGGGGTACCATGGAGAAAACA GAATCACCA-3′, the *Kpn*I site) and (Reverse, 5′-CGGGATCCCGTGAAGGTTTTGGCGGAA-3′, the *BamH*I site). The resulting construct was introduced into *Populus deltoides × P. euramericana* cv “nanlin895” *via* leaf disc method (Chai et al., 2014). The transgenic poplar plantlets were selected on a medium containing 5 mg L^−1^ hygromycin and identified through PCR at the DNA level and qRT‐PCR at the mRNA level. PCR primers: 35Spro: ATGACGCACAATCCCACTATCC; *Pd*C3H17R: 5′-CAGTGAGGGAGTGGCGAAAG-3′; *Pd*C3H17ΔCR: 5′-TCGTTTGCATTAAACACCTCCA-3′. qRT-PCR primers: *Pd*C3H17 (Forward: 5′-CAAGTGGCAAGAGACAGGCA-3′; Reverse: 5′-CAGTGAGGGAGTGGCGAAAG-3′) and *Pd*C3H17ΔC (Forward: 5′-ACTCAACTCGGTCTCGTGTG-3′; Reverse: 5′-TCGTTTGCATTAAACACCTCCA-3′). Regenerated plantlets were acclimatized in a mist chamber for 30 days, and transferred to a greenhouse with a 16/8 h light/dark photoperiod at 25°C to 28°C.

### Quantitative Real-Time RT-PCR (qRT-PCR)

Total RNA isolation and first-strand cDNA synthesis were performed as described previously (Chai et al., 2014). The qRT-PCR assays were conducted on a LightCycler^®^480 Detection System (Roche) using a SYBR Premix Ex Taq II (TaKaRa) kit. The expression was normalized using reference gene *PdUBQ* (BU879229, Forward, 5′-GTTGATTTTTGCTGGGAAGC-3′; Reverse, 5′-GATCTTGGCCTTCACGTTGT-3′), and determined by the 2^−ΔΔCT^ method (Livak and Schmittgen, 2001). Data represent the average of at least three biological replicates.

### Microscopy

Cross section of the basal stems was produced from 3-month-old transgenic poplars as described previously (Chai et al., 2014). For each construct, at least six plants of two lines were examined. Briefly, 0.5-cm stem segments were submerged in 4% paraformaldehyde for 3 days, then dehydrated in a graded ethanol series, and finally incubated in pure paraplast. The paraplast-embedded stems were sectioned to a thickness of 7 μm using a Leica RM 2235 microtome (Leica) and adhered to Superfrost Plus microscope slides (Thermo Fisher). Stem sections were stained with Toluidine Blue-O (TBO, 1% w:v) and then observed using an Olympus DX51 microscope.

### Drought Treatment

Three-month-old transgenic poplar lines encountered drought treatment, in which the soil RWC was reduced from 70% following described previously (Wang et al., 2016). Control plants were kept in the same conditions, except that the soil RWC was maintained at 70%. Control and transgenic plants were grown in suitably sized pots, and each pot had a tray. Photographs were taken after treatment for 20 days.

### Measurement of Physiologic Parameters

At 20th day of drought treatment, net photosynthetic rate, stomatal conductance, transpiration, and chlorophyll a+b content were detected in the 5th to 7th leaves of control and transgenic lines using Li-6400 Photosynthesis System (Li-Cor Biosciences, Lincoln, NE). The 4th to 6th leaves from the top of the plants were sampled for measurement of the amounts of malondialdehyde (MDA), hydrogen peroxide (H_2_O_2_), four ROS-scavenging enzymes (superoxide dismutase, SOD; peroxidase, POD; ascorbate peroxidase, APX; and catalase, CAT) and three osmotic adjustments (proline, soluble protein, and soluble sugar). At least five biological replicates were performed for each genotype. The levels of H_2_O_2_, SOD, POD, APX, CAT, and soluble protein were measured according to the methods described by Shi et al. (2014).

The levels of MDA, proline, and soluble sugar were detected with the commercial kits following the instructions (Nanjing Jiancheng Bioengineering Institute, China). For determination of MDA content, 0.1 g of sample was extracted with 1 mL of 20% (w/v) trichloroacetic acid (TCA). The homogenate was centrifuged at 3500*g* for 20 min, 2 mL supernatant was added to 2 mL of 20% TCA containing 0.5% (w/v) triobarbituric acid (TBA). After heating in 95°C for 30 min and cooling in ice bath, the proline level was calculated based on absorbance at 532 and 600 nm. The value for non-specific absorption at 600 nm was subtracted from the value at 532 nm. For determination of proline content, 0.1 g of sample was ground and extracted in 3% (w/v) sulphosalicylic acid. After reacting with acid ninhydrin solution, the proline level was calculated based on absorbance at 520 nm. For the determination of soluble sugar content, 0.1 g of sample was extracted in 5 ml of 80% (v/v) ethanol at 80 C for 40 min and centrifuged at 12000 rpm for 10 min. The supernatants were dipigmented by litter activated charcoal at 80°C for 30 min. The mixture of 0.1 ml of the extracts and 3 ml of 0.15% (w/v) anthrone reagent (0.3 g anthrone was dissolved in 200 ml of 7.74 M H_2_SO4) was heated at 90°C for 20 min. The level of soluble sugar was examined at 620 nm of absorbance by making the specification curve with known concentration of glucose.

Water potential was measured under well-watered condition or with drought treatment for 20 days. For each genotype, at least six plants of two lines were selected. A TP-PW-II Water Potential System (TPYN SciTech, Hangzhou, China) was used for the measurement of stem water potential according to the manufacturer's instructions. Statistical analyses were performed based on data from two independent experiments.

### Statistical Analysis

All data were presented as mean ± standard errors. The statistical significance of differences between data was evaluated using one-way analysis of variance (ANOVA) followed by Duncan's multiple range test (*P* < 0.05).

## Results

### Transcriptional Activation Analysis of *Pd*C3H17

Our previous study showed that *Populus Pd*C3H17 functions as positive regulators of secondary xylem development in both *Arabidopsis* and poplar (Chai et al., 2014). To elucidate the transcriptional properties of *Pd*C3H17, multiple fusion proteins were constructed, in which the coding region and selected portions of *Pd*C3H17 were fused to the GAL4 DNA-binding domain. All of the transformed yeast cells grew well on SD/Trp-medium. Transcription of the *HIS* reporter gene was significantly activated by GAL4-*Pd*C3H17 N-terminal region that excludes the CCCH domain (ΔC), but not by the GAL4-full-length *Pd*C3H17 protein (FL) and the GAL4-CCCH domain (ΔN) (**Figure 1**). These results indicated that the N-terminal sequence of *Pd*C3H17 may possess transcriptional activation capacity.

### Phenotypes of Transgenic Poplars Overexpressing *PdC3H17 or PdC3H17ΔC*

**Figure 2:**
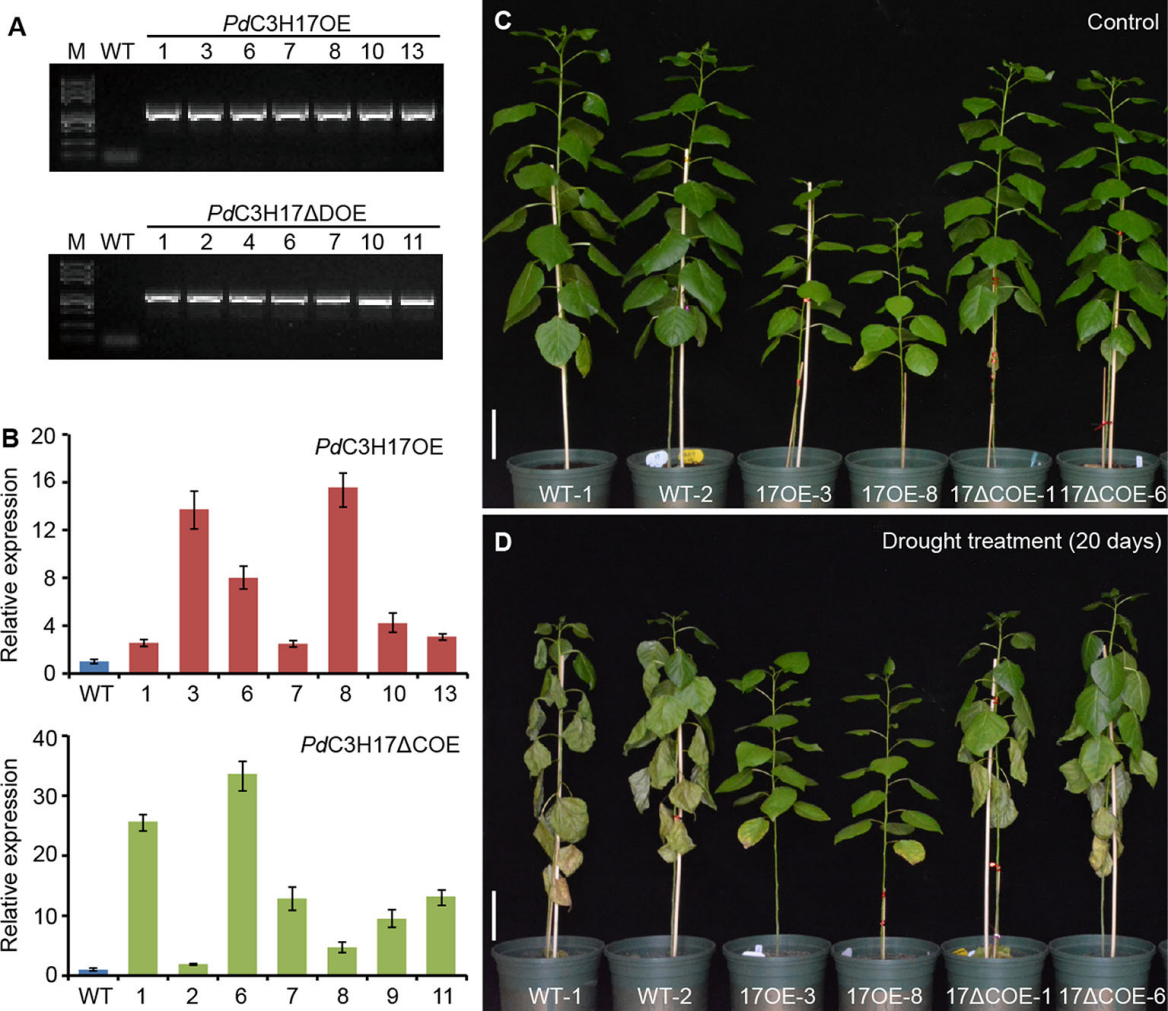
Morphological differences between wild-type and transgenic poplars overexpressing *PdC3H17* or *PdC3H17ΔC* after well-watered or drought stress conditions for 20 days. **(A)** PCR identification of representative *PdC3H17* and *PdC3H17ΔC* overexpression poplar lines using *pro35S* and gene-specific primers. Wild-type (WT) plants were used as the control. **(B)** qRT-PCR analysis of *PdC3H17* expression in WT, *Pd*C3H17OE, or *Pd*C3H17ΔCOE poplar lines. **(C, D)** Drought response of 3-month-old representative WT, *Pd*C3H17OE (17OE), and *Pd*C3H17ΔCOE (17ΔCOE) poplar lines at the 20th day of well-watered **(C)** or drought **(D)** treatment. Bars = 10 cm.

The structure and size of xylem vessel cells are key factors affecting water transport in plants and are important determinants of drought tolerance (Fisher et al., 2007; Li et al., 2019). To investigate whether *Pd*C3H17 participates in controlling drought response, we generated transgenic poplar lines overexpressing the full length coding region (*Pd*C3H17OE) or the fragment excluding the CCCH domain (*Pd*C3H17ΔCOE) of *PdC3H17* (**Figure 1A**). For each construct, at least 30 independent transgenic lines were generated and two lines with highest expression of the transgene were selected for further characterization (**Figures 2A, B**). Consistent with our previous phenotypic observation (Chai et al., 2014), *Pd*C3H17OE lines exhibited dwarf compared with WT controls (**Figure 2C**). Considering that AtC3H14 and AtC3H15 redundantly control cell elongation in stems (Kim et al., 2014; Chai et al., 2015), we suggest that *Pd*C3H17 may play a negative role in controlling stem elongation. Interestingly, we found that *Pd*C3H17ΔCOE lines roughly showed wild type-like phenotypes (**Figure 2**), suggesting that overexpression of *PdC3H17* inhibits stem elongation likely depending on its CCCH domain.

### Different Drought Responses of *PdC3H17* and *PdC3H17ΔC* Overexpression Poplars Are Associated With the Changes of Their Stem Xylem Vessel Cell Number

To determine the biological role of *PdC3H17* in drought stress, WT, *Pd*C3H17OE, and *PdC3H17*ΔCOE plants grown in soil for 3 months were exposed to drought treatment by withholding water. Most leaves of the WT poplars were seriously wilted on day 20, whereas those of *Pa*C3H17OE plants remained normal (**Figure 2D**). The drought response of *PdC3H17*ΔCOE plants was slightly stronger than that of WT plants but significantly weaker than that of *Pd*C3H17OE plants. Under the normally watered condition, WT and *PdC3H17*ΔCOE plants remained drastically faster growth than *PdC3H17*OE plants (**Figure 2C**).

**Figure 3:**
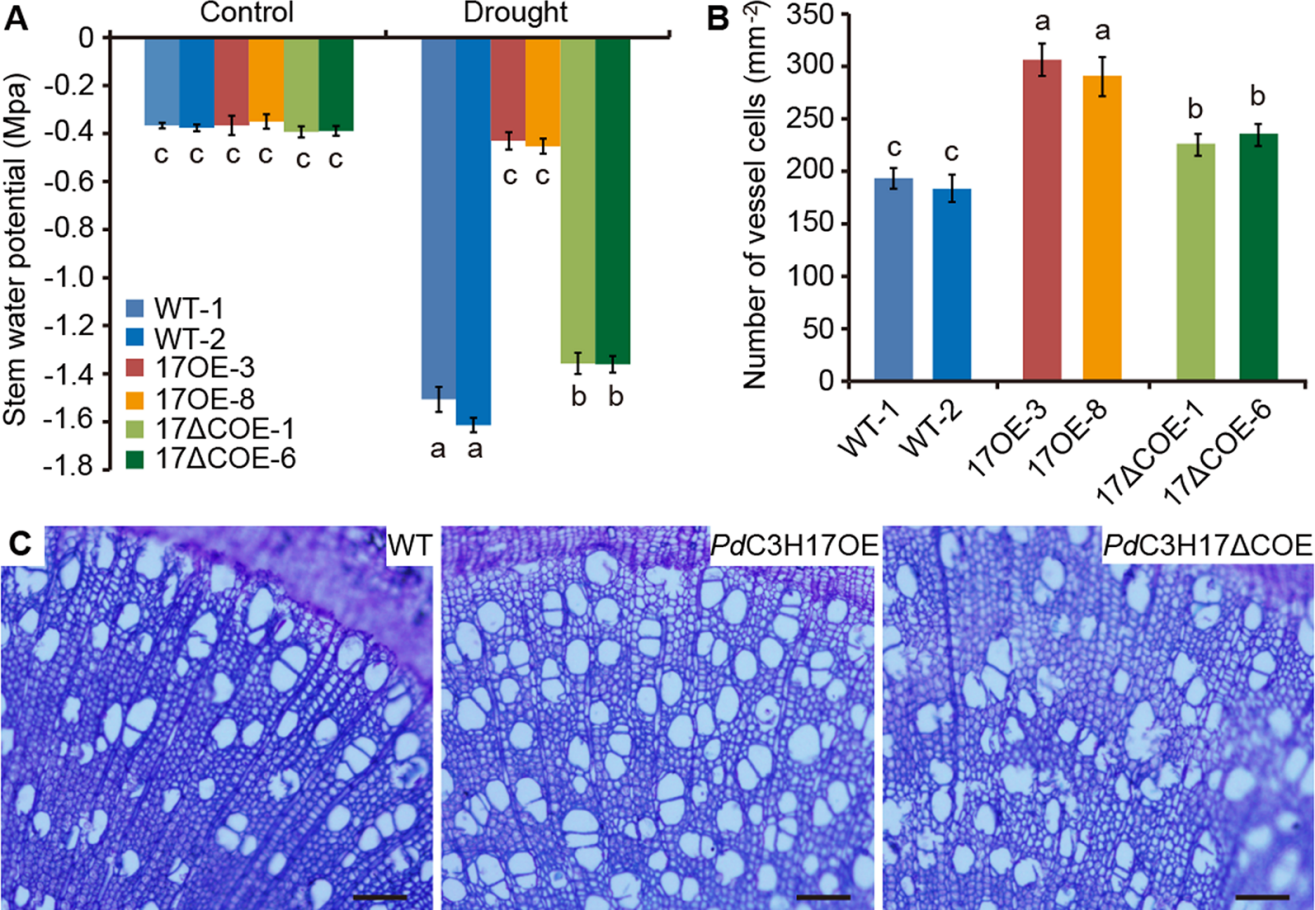
Stem water potential and xylem vessel cell numbers of WT, *Pd*C3H17OE, and *Pd*C3H17ΔCOE poplars. **(A)** Statistical analysis of stem water potential of 4-month-old WT, *Pd*C3H17OE (17OE), and *Pd*C3H17ΔCOE (17ΔCOE) poplars under well-watered and drought stress conditions for 20 days. **(B)** Statistical analysis of the numbers of stem xylem vessel cells in 3-month-old WT, 17OE, and 17ΔCOE poplars. At least six plants from two lines in each genotype were selected for measurement of stem water potential and xylem vessel cells. Data are presented as mean ± SD. Different letters above bars denote statistical significance between treatments of samples (*P* < 0.05). **(C)** Basal stem sections of 3-month-old representative WT, *Pd*C3H17OE, and *Pd*C3H17ΔCOE plants. Bars = 100 μm.

We further investigated the effects of *PdC3H17* or *PdC3H17ΔC* overexpression on alterations in physiology that may contribute to drought response. It is known that higher stem xylem water potential can prevent drought-induced hydraulic failure and enhance drought resistance (Choat et al., 2012). Our results indicated that stem xylem water potential was the highest in *Pd*C3H17OE plants, followed in *Pd*C3H17*ΔC* OE plants, and the lowest in WT plants under drought stress (**Figure 3A**), which was consistent with their visible phenotypes (**Figure 2D**). By contrast, the three genotypes showed similar stem water potential under control condition (**Figure 3A**). We then analyzed the morphology of stem xylem cells. As indicated in **Figures 3B, C** the vessel number per unit of area was the greatest in *Pd*C3H17OE plants, following in *Pd*C3H17ΔCOE plants, and the smallest in WT plants. No significant difference for cell size of stem xylem vessels was observed between the three genotypes. It is possible that the increase of stem vessel cell number in *Pd*C3H17 and *Pd*C3H17ΔC overexpression poplars contributes to more effective water transport, thereby promoting drought tolerance.

### Photosynthetic Capacities Are Differentially Repressed in *PdC3H17* and *PdC3H17ΔC* Overexpression Poplars After Drought Treatment

**Figure 4:**
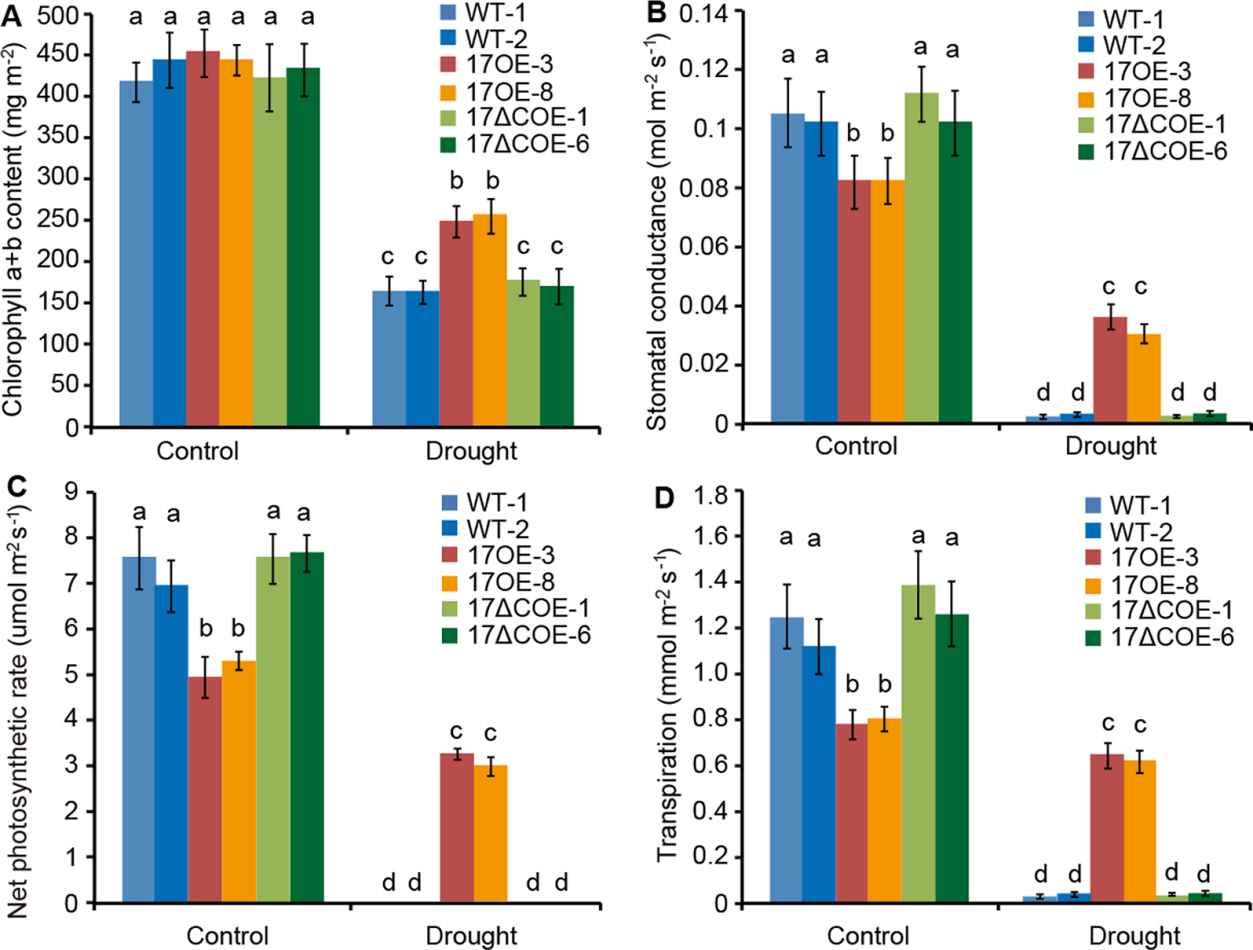
Variation in photosynthetic parameters of 3-month-old *Pd*C3H17OE (17OE) and *Pd*C3H17ΔCOE (17ΔCOE) plants relative to those of WT plants after well-watered or drought stress conditions for 20 days. **(A)** Chlorophyll a+b content. **(B)** Stomatal conductance. **(C)** Net photosynthetic rate. **(D)** Transpiration. At least six plants of two lines in each genotype were measured. Data are presented as mean ± SD. Different letters above bars denote statistical significance between treatments of leaves (*P* < 0.05).

Four photosynthetic parameters (chlorophyll a+b content, stomatal conductance, net photosynthetic rate, and transpiration) were detected in WT, *PdC3H17*OE, and *PdC3H17*ΔCOE poplars (**Figure 4**). At 20th day of normal-watered condition, chlorophyll a+b content was roughly similar among three genotypes (**Figure 4A**). However, stomatal conductance, transpiration, and net photosynthetic rate in *Pd*C3H17OE plants were markedly lower than those in WT and *PdC3H17*ΔCOE plants (**Figures 4B–D**). When treated by drought stress for 20 days, four photosynthetic parameters were decreased in all detected plants, but these decreases were more obvious in both WT and *PdC3H17*ΔCOE plants than in *Pd*C3H17OE plants (**Figure 4**). These results suggest that overexpression of *PdC3H17* increases drought tolerance likely depending on its CCCH domain.

### The ROS-Scavenging Abilities Are Differentially Increased in *PdC3H17* and *PdC3H17ΔC* Overexpression Poplars After Drought Treatment

**Figure 5:**
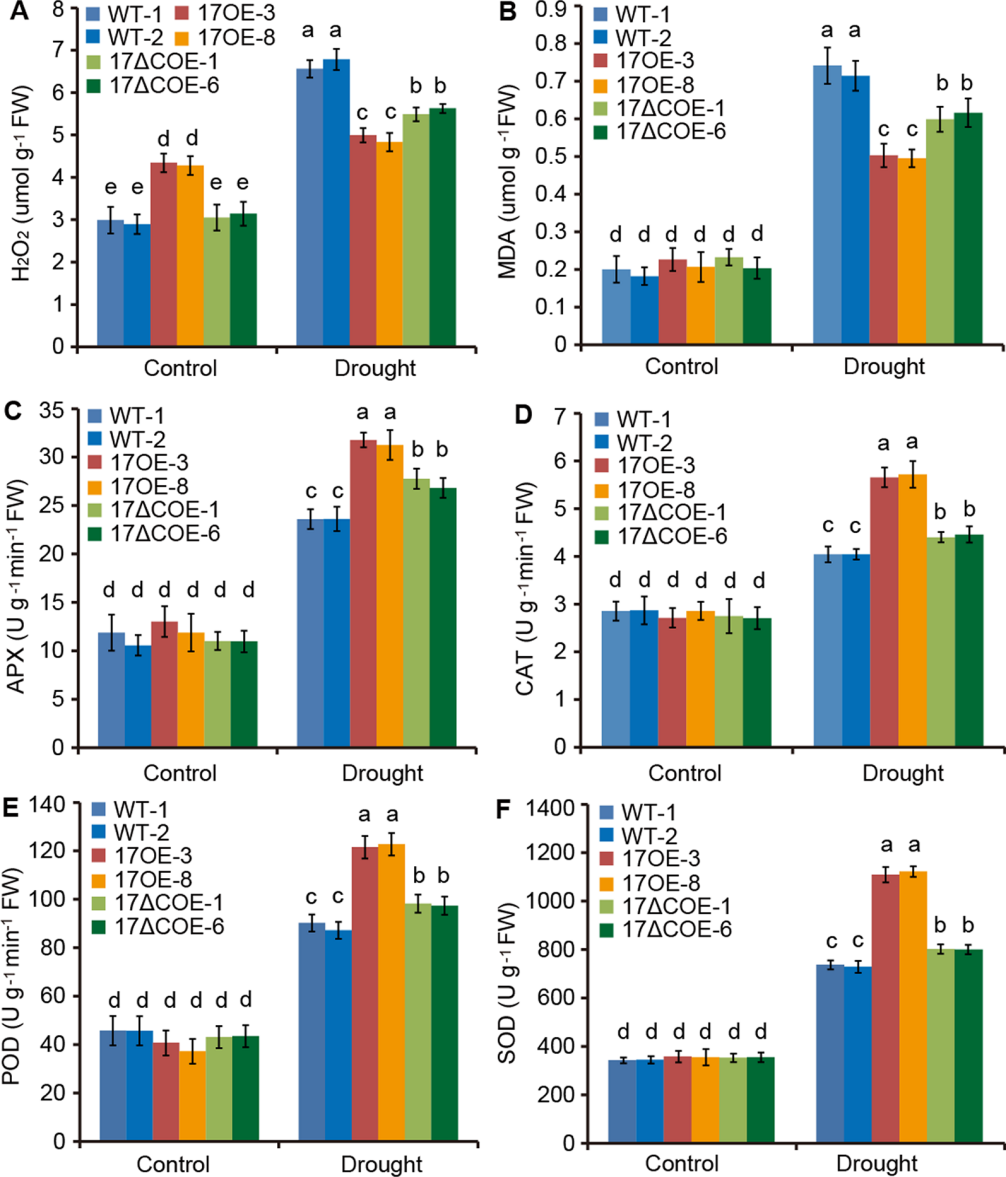
Quantitative analyses of H_2_O_2_ and four antioxidants in 3-month-old WT, *Pd*C3H17OE (17OE), and *Pd*C3H17ΔCOE (17ΔCOE) poplars after well-watered or drought stress conditions for 20 days. **(A)** H_2_O_2_, hydrogen peroxide. **(B)** MDA, malondialdehyde. **(C)** APX, ascorbate peroxidase. **(D)** CAT, catalase. **(E)** POD, peroxidase. **(F)** SOD, superoxide dismutase. At least six plants of two lines in each genotype were measured. Data are presented as mean ± SD. Different letters above bars denote statistical significance between treatments of leaves (*P* < 0.05).

ROS play a key role in the acclimation process of plants to drought stress (Choudhury et al., 2017). We thus examined whether overexpression of *PdC3H17* or *PdC3H17ΔC* affects the accumulation of ROS in transgenic poplars. The levels of H_2_O_2_, a major ROS, were slightly higher in *Pd*C3H17OE plants than in WT and *Pd*C3H17ΔCOE plants under control condition. However, at 20 days of drought stress a lower accumulation of H_2_O_2_ was observed in *Pd*C3H17OE plants relative to other two genotypes (**Figure 5A**). Further, the *Pd*C3H17ΔCOE plants showed less increase of H_2_O_2_ level than WT plants. These results indicated that overexpression of *PdC3H17* and *PdC3H17ΔC* in poplar differentially affected drought-induced ROS accumulation. Similarly, the increased levels of MDA, an indicator of cytomembrane oxidative damage (Shi et al., 2014), were the highest in WT plans and the lowest in *Pd*C3H17OE plants after drought treatment (**Figure 5B**), suggesting that WT plants may undergo more serious membrane damage than these transgenic plants.

**Figure 6:**
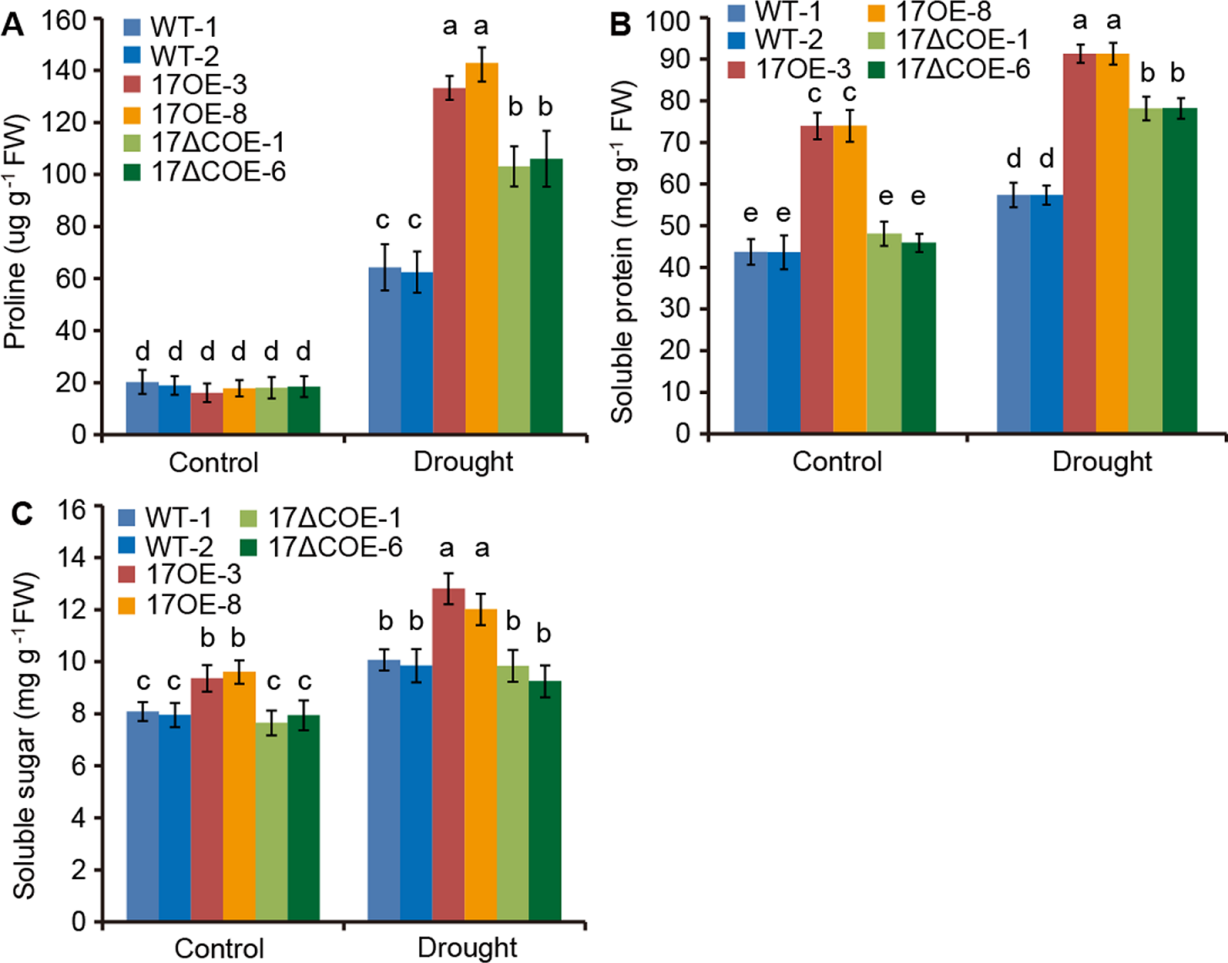
Quantitative analyses of osmotic adjustment substances in 3-month-old WT, *Pd*C3H17OE (17OE), and *Pd*C3H17ΔCOE (17ΔCOE) poplars after well-watered or drought stress conditions for 20 days. **(A)** Free proline. **(B)** Soluble protein. **(C)** Soluble sugar. At least six plants of two lines in each genotype were measured. Data are presented as mean ± SD. Different letters above bars denote statistical significance between treatments of leaves (*P* < 0.05). *P* < 0.05.

6APX, CAT, POD, and SOD are crucial antioxidants that can scavenge ROS (Choudhury et al., 2017). We further detected the activities of these four enzymes in drought-treated and control WT and transgenic poplars. Corresponded with the H_2_O_2_ levels, the activities of APX, CAT, POD, and SOD were most obviously elevated in *Pd*C3H17OE plants, following *Pd*C3H17ΔCOE plants, and less significantly increased in WT plants when treated by drought (**Figures 5C–F**). Besides, free proline, soluble protein, and soluble sugar contents were measured in these poplars (**Figures 6A–C**). Under control condition, *Pd*C3H17OE plants showed higher levels of soluble protein and soluble sugar than WT and *Pd*C3H17ΔCOE plants (**Figures 6B–C**). After drought stress for 20 days, free proline, soluble protein, and soluble sugar were more obviously accumulated in *Pd*C3H17OE plants than in other two genotypes (**Figures 6A–C**). These data indicated that *PdC3H17* overexpression may enhance the ROS scavenging capacity and thereby confer plant resistance to drought stress.

### Weaker Growth Inhibition is Observed in *PdC3H17* Overexpression Poplars During Drought Stress

**Figure 7:**
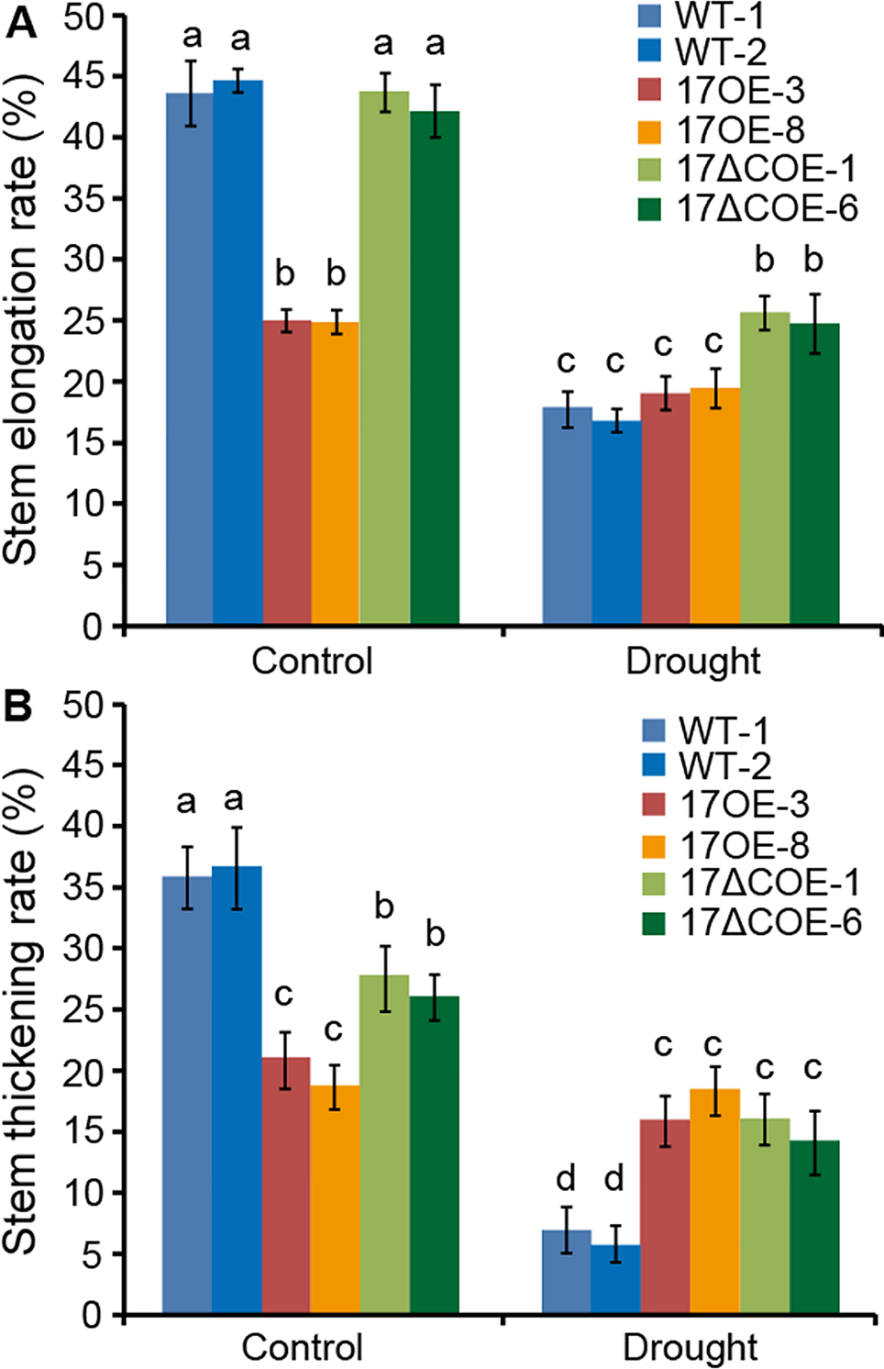
Stem elongation and thickening rates of 3-month-old WT, *Pd*C3H17OE (17OE), and *Pd*C3H17ΔCOE (17ΔCOE) poplars after well-watered or drought stress conditions for 20 days. **(A)** Stem elongation rate. **(B)** Stem thickening rate. At least six plants of two lines in each genotype were measured. Data are presented as mean ± Different letters above bars denote statistical significance between treatments of samples (*P* < 0.05).

We further investigate whether overexpression of *PdC3H17* or *PdC3H17ΔC* in poplar affects stem growth in the absence of water stress. Statistical analyses revealed that drought treatment resulted in a significantly greater decrease of both stem elongation and thickening rates in WT and *Pd*C3H17ΔCOE poplars than in *Pd*C3H17OE poplars compared to control condition (**Figure 7**). Furthermore, these decreases were more obvious in WT plants than in *Pd*C3H17ΔCOE plants. Therefore, *Pd*C3H17OE poplars maintained higher growth than WT plants under long-term water deficit conditions.

## Discussion

Drought is the primary abiotic stress responsible for inhibiting poplar growth (Yin et al., 2004; Monclus et al., 2006; Harfouche et al., 2014). Clarifying the regulatory mechanisms of drought tolerance in poplar is essential for ecological conservation and wood production. In this study, we provide evidence showing that *Pd*C3H17 is a novel regulator of drought response and tolerance. Overexpression of *PdC3H17* in a hybrid poplar resulted in a significant increase in drought tolerance in comparison with WT plants, correlating with visible alterations of their physiological parameters. As far as we know, this is the first report of CCCH-mediated drought response in woody species.

Wood is made up of xylem, the conductive tissue that transports water from soil to leaves and provides mechanical support for the entire plant (Evert, 2006). Hydraulic conductivity in xylem is highly related to cell size and number of stem xylem vessels (Fisher et al., 2007; Li et al., 2019). For instance, transgenic poplars overexpressing *PtrNAC006*, *PtrNAC007*, or *PtrNAC120* have smaller vessel lumen area and more vessel cells than WT plants, resulting in stronger drought tolerance (Li et al., 2019). Here, our observations of stem sections revealed that the *Pd*C3H17OE poplars had more xylem vessel cells than WT controls, correlating with higher stem water potential in these transgenic plants (**Figure 3**). These may result in stronger stem hydraulic conductance in *Pd*C3H17OE plants than in WT plants. Stress-resistant plants often have a dwarf form (Hu et al., 2005; Yu et al., 2008). Because the dwarf is benefit for reducing water and energy consumption and facilitates energy redistribution. Our current results revealed that non-stressed *Pd*C3H17OE poplars showed growth inhibition phenotypes with reduced plant height and decreased stomatal conductance, transpiration, and net photosynthetic rate (**Figures 2** and **4**). Further, *Pd*C3H17OE lines showed higher stem elongation and thickening rates than WT plants under water‐deficit condition (**Figure 7**). Therefore, *Pd*C3H17OE poplars enhance drought tolerance likely due to greater hydraulic conductance and dwarf phenotype.

H_2_O_2_ is one of the major ROS in plants, and its level is significantly increased under stress conditions (Bhattacharjee, 2012; Choudhury et al., 2017). To minimize oxidative damage, plants have developed sophisticated ROS-scavenging mechanisms. Here, we found that drought stress led to mass accumulation of H_2_O_2_ in all detected poplars (**Figure 5A**). However, the accumulation of H_2_O_2_ in WT plants was obviously higher than that in *Pd*C3H17OE plants (**Figure 5A**), and meanwhile the stomatal conductance and photosynthesis in the WT lines almost reached zero (**Figures 4B, C**). Accordingly, the activities of four major ROS-scavenging enzymes (APX, CAT, POD, and SOD) were increased less significantly in WT plants than in *Pd*C3H17OE plants after drought treatment (**Figures 5C–F**). Thus, another possible reason for strong drought tolerance of *Pd*C3H17OE poplars may be more effective activation of the antioxidant system.

Tristetraprolin (hTTP) and AtC3H14 are the orthologs of *Pd*C3H17 in human and *Arabidopsis*, respectively (Chai et al., 2012). The two proteins have the DNA/RNA binding abilities and may perform transcriptional regulation in the nucleus and RNA regulation in cytoplasm (Lai et al., 1999; Kim et al., 2014; Chai et al., 2015). The best-known CCCH protein is human hTTP, which binds to AU-rich elements (AREs; AUUUA) in the 3′-untranslated region (UTR) of target genes such as TNF-alpha, and performs post-transcriptional regulation (Lai et al., 1999). Further, the CCCH domain of hTTP is necessary for the deadenylation and degradation of target mRNAs in cytoplasmic foci (Lai et al., 1999; Michel et al., 2003). RNA-EMSA data showed that AtC3H14 from *Arabidopsis* also requires its CCCH domain for target RNA binding (Kim et al., 2014). Our current results revealed that *Pd*C3H17OE poplars exhibited dwarf and drought-tolerance phenotypes whereas morphology and drought response of *Pd*C3H17ΔCOE poplars are more similar to WT plants (**Figure 2**), suggesting that overexpression of *PdC3H17* in a hybrid poplar may confer drought tolerance and inhibit stem elongation depending of its CCCH domain. Considering that *Pd*C3H17, like hTTP and AtC3H14, shares typical CX_8_CX_5_CX_3_H motifs and is targeted to cytoplasmic foci (Chai et al., 2014), we speculate that *Pd*C3H17 might function in drought tolerance and stem elongation through CCCH domain-dependent and post-transcriptional regulation in *Populus*. Further studies need to be conducted to resolve these ambiguities. Interestingly, we found that the N-terminal sequence of *Pd*C3H17, but not full-length *Pd*C3H17, had transcriptional activation ability in yeast cells (**Figure 1**). LIC, a CCCH protein in rice, is shown to function as a negative regulator of the Brassinosteroid (BR) signaling pathway through direct suppression of BZR1 targets (Zhang et al., 2012). Thus, it is possible that *Pd*C3H17, like LIC, acts as a transcriptional repressor of stem development and stress resistance, in addition to functioning at the post-transcriptional level. This hypothesis needs to be further validated experimentally.

In conclusion, we developed transgenic *PdC3H17-* or *PdC3H17ΔC*-expressing poplar plants. *Pd*C3H17OE poplar was slightly dwarf, had more stem xylem vessel cells, and enhanced ROS-scavenging abilities, thereby reinforcing plant tolerance to drought stress. In contrast, *Pd*C3H17ΔCOE poplar showed WT-like phenotypes, indicating the requirement of its CCCH domain for drought response. Our previous study demonstrated that *Pd*C3H17OE poplars had significantly wider xylem and thicker secondary cell walls than WT controls after growth of 4 months (Chai et al., 2014). Therefore, *Pd*C3H17 may have potential for use in the genetic improvement of drought tolerance and wood production in *Populus*.

## Data Availability Statement

The datasets generated for this study are available on request to the corresponding authors.

## Author Contributions

GC designed the experiment, performed data processing, and drafted the manuscript. YZhu, CW, YZha, SC, DW, and QL prepared the materials and performed the experiments. GZ conceived the study and revised the manuscript. All authors read and approved the final version of the manuscript.

## Funding

Financial support for this work was obtained from National Key Scientific Research Project of China (2016YFD0600104), National Key Program on Transgenic Research (2018ZX08020002), National Natural Science Foundation of China (31670606, 31570670, 31770315, and 31700526), Major Basic Research Project of Shandong Natural Science Foundation (ZR2018ZC0335), Shandong Provincial Natural Science Foundation (ZR2017BC096, ZR2017BC078, and ZR2019BC006), and Taishan Scholar Program of Shandong (to GZ).

## Conflict of Interest

The authors declare that the research was conducted in the absence of any commercial or financial relationships that could be construed as a potential conflict of interest.
